# Unraveling the dysregulation of Frizzled receptors in Alzheimer’s disease: Critical contributor for synapse loss

**DOI:** 10.1002/alz.090214

**Published:** 2025-01-03

**Authors:** Nimisha Basavaraju, Reddy Peera Kommaddi

**Affiliations:** ^1^ Centre for Brain Research, Indian Institute of Science, Bangalore, Karnataka India; ^2^ Manipal Academy of Higher Education, Manipal, Karnataka India; ^3^ Centre for Brain Research, Bangalore, Karnataka India

## Abstract

**Background:**

Alzheimer’s disease (AD) is characterized by early synapse loss, which is further associated with deficits in memory and cognition. The loss of synapses could be mediated by dysregulation of molecular mechanisms crucial for maintaining synaptic structure and function. Among these mechanisms, the Wnt signaling pathway holds significant importance in regulating synaptic assembly. Specifically, Frizzled receptors have been identified as necessary and/or sufficient to recruit synaptic proteins at both the pre‐ and post‐synapse. Despite the pivotal role of frizzled receptors at the synapse, their contribution to synapse loss in AD remains relatively unclear. This lack of clarity persists as existing research on dysregulation of Wnt signaling in AD has predominantly focused on antagonist (Dkk1), kinase (Gsk3β), and co‐receptor (Lrp6). So, the goal of our study is to unravel how frizzled receptors are regulated and how they affect the phosphorylation and ubiquitination of β‐catenin at the synapse in AD.

**Method:**

Post nuclear supernatant and synaptosomes were isolated from brain cortex of nine‐month‐old male wild type and APP/PS1 mice. The samples were subjected to immunoblotting against Frizzled receptor 7 (Fzd7) and phosphorylation of β‐catenin. β‐catenin immunoprecipitated samples of synaptosomes were immunoblotted and probed for β‐catenin ubiquitination. Statistical comparisons between wild type and APP/PS1 group were conducted using two tailed, unpaired Mann‐Whitney *U* test.

**Result:**

Protein levels of Fzd7 are significantly decreased in synaptosomes of nine‐month‐old APP/PS1 mice compared to that of control mice. Decrease in the levels of receptor, Fzd7 implies an increase in β‐catenin phosphorylation and ubiquitination at the synaptosomes as a result of decreased Wnt signaling. Intriguingly, our observations reveal a significant decrease in both phosphorylation and ubiquitination of β‐catenin in synaptosomes of nine‐month‐old APP/PS1 mice.

**Conclusion:**

Decreased phosphorylation and ubiquitination of β‐catenin is observed in nine‐ month‐old APP/PS1 mice compared to wild type suggesting an elevated Wnt signaling at the synapse. Our study provides novel insight into how elevated Wnt signaling could contribute to the synapse loss in AD.

